# Correction: Seed dressing with mefenpyr-diethyl as a safener for mesosulfuron-methyl application in wheat: The evaluation and mechanisms

**DOI:** 10.1371/journal.pone.0314903

**Published:** 2024-12-02

**Authors:** The PLOS One Staff

There is an error in affiliation 2 for authors Guangyuan Ma, Yaling Geng, Hua Wang, Jian Li, Shanshan Song, Wenliang Pan, Zhiying Hun. The correct affiliation 2 is: Plant Protection Institute, Hebei Academy of Agricultural and Forestry Sciences/ IPM Centre of Hebei Province/ Key Laboratory of Integrated Pest Management on Crops in Northern Region of North China, Ministry of Agriculture and Rural Affairs, Baoding, Hebei, China.

The publisher apologizes for the error.
