# Supplementary figures and images for: Seed dressing with mefenpyr-diethyl as a safener for mesosulfuron-methyl application in wheat: The evaluation and mechanisms
Source: PLoS One. 2021 Aug 30;16(8):e0256884. doi: 10.1371/journal.pone.0256884 (PMC8405001; doi:10.1371/journal.pone.0256884)

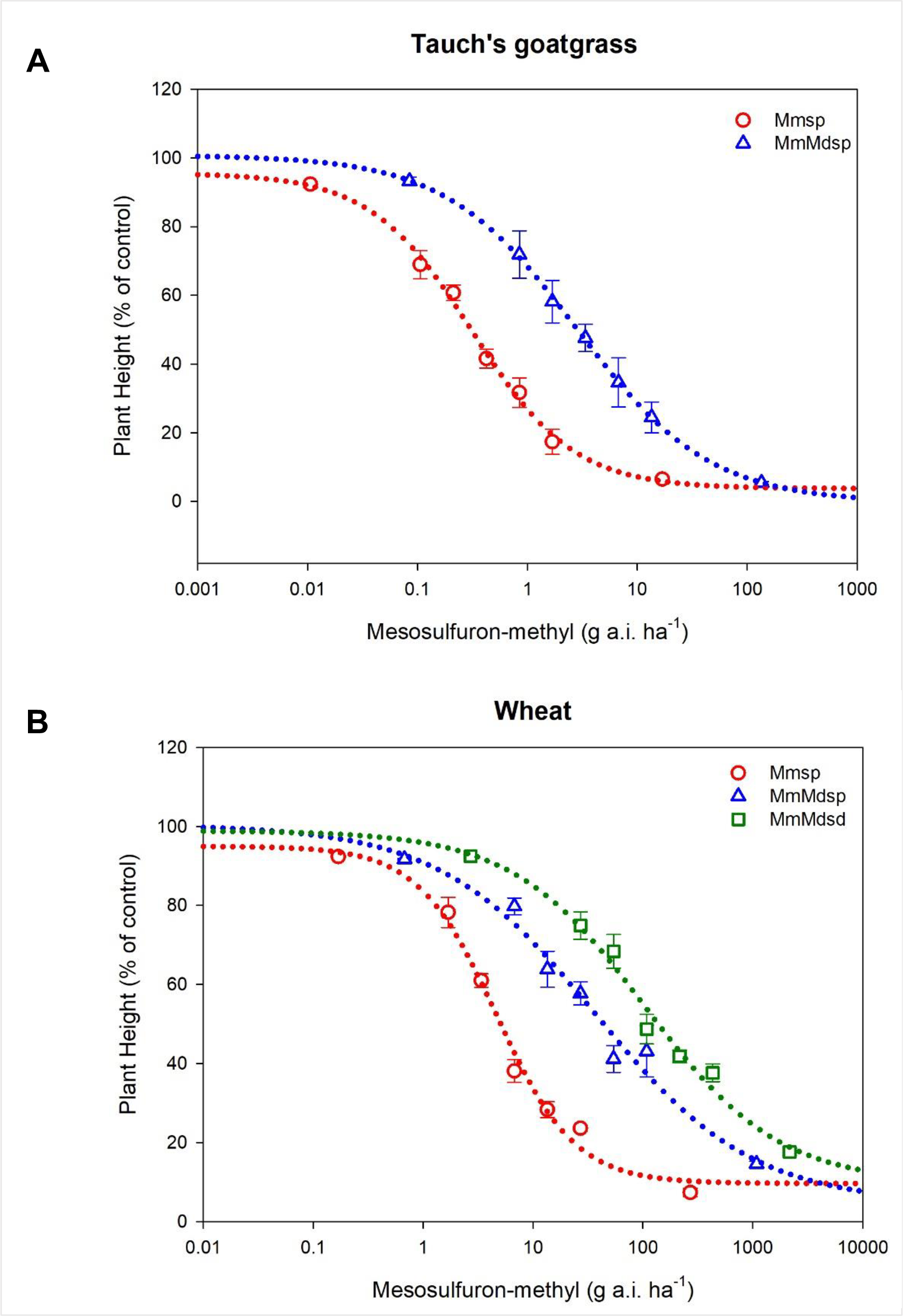

Supplement: S1 Fig — Dose-response curve of Tausch’s goatgrass (A) and wheat (B) treated with different doses of mesosulfuron-methyl with or without mefenpyr-diethyl applied by spraying or seed dressing. Plant height was expressed as a percentage of the untreated control. Each data point represents the mean ± SE of twice-repeated experiments containing three replicates each, and vertical bars represent the standard error. Mmsp: Plants treated with mesosulfuron-methyl by spraying, MmMdsp: Plants treated with mesosulfuron-methyl and mefenpyr-diethyl by spraying, MmMdsd: Plants treated with Mesosulfuron-methyl by spaying and pretreated with mefenpyr-diethyl by seed dressing. (TIF) [file pone.0256884.s001.tif]

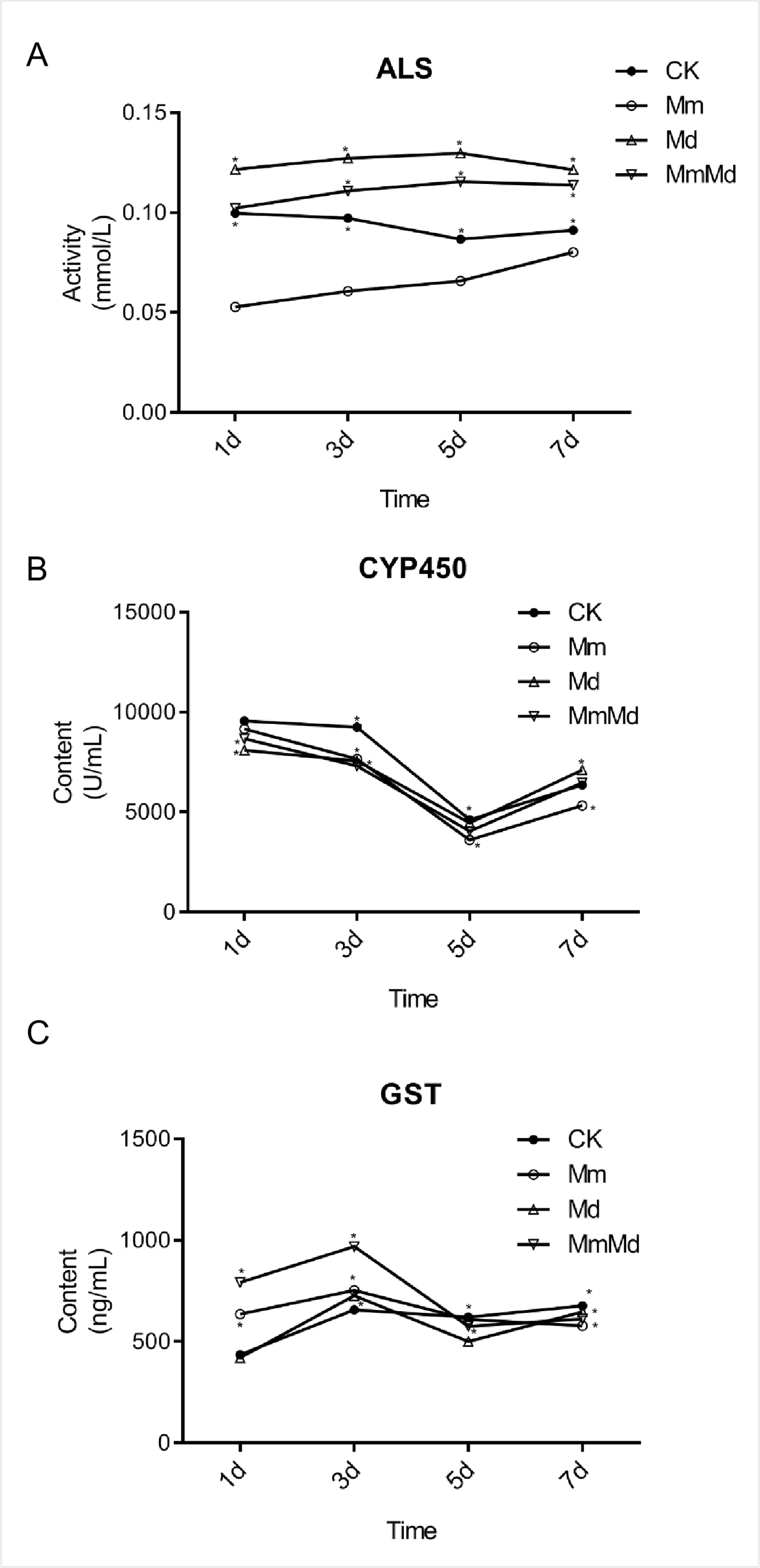

Supplement: S2 Fig — Effect of mesosulfuron-methyl and/or mefenpyr-diethyl on the ALS activity (A), and CYP450 (B) and GST(C) content. Error bars indicate standard errors. *Significantly different at the P < 0.05 level compared to CK. (TIF) [file pone.0256884.s002.tif]

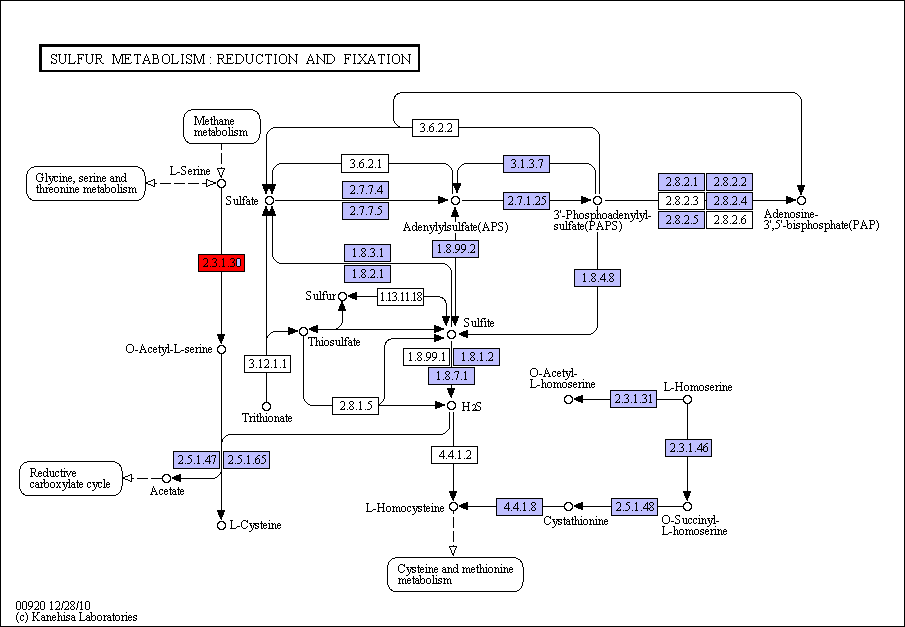

Supplement: S3 Fig — (TIF) [file pone.0256884.s003.tif]
