# Supplementary material for: Seed dressing with mefenpyr-diethyl as a safener for mesosulfuron-methyl application in wheat: The evaluation and mechanisms
Source: PLoS One. 2021 Aug 30;16(8):e0256884. doi: 10.1371/journal.pone.0256884 (PMC8405001; doi:10.1371/journal.pone.0256884)
Supplement: S1 Table — (DOCX) [file pone.0256884.s004.docx]

| Dose  (g per kg seed) | Emergence rate  (%) | leaf length (cm) | | |
| --- | --- | --- | --- | --- |
|  |  | 1^st^ leaf | 2^nd^ leaf | 3^rd^ leaf |
| 0.00 | 97.50±0.03 | 9.02±0.16 | 13.81±0.31 | 10.57±0.47 |
| 0.50 | 97.50±0.03 | 8.64±0.15 | 12.98±0.16 | 10.24±0.09 |
| 1.00 | 97.50±0.03 | 8.74±0.17 | 12.97±0.29 | 10.21±0.31 |
| 2.00 | 95.00±0.05 | 8.76±0.22 | 13.31±0.24 | 10.10±0.22 |
| 4.00 | 92.50±0.03 | 8.10±0.22* | 12.40±0.28* | 10.68±0.70 |
| 8.00 | 82.50±0.05* | 7.46±0.34* | 12.06±0.60* | 10.93±0.62 |

**S1 Table. Growth response of wheat treated by seed dressing with different mefenpyr-diethyl application doses.**

Data are shown as mean ± SE.

* indicates significant difference (*P* < 0.05) between treatments and control (0 g per kg seed)
