# Supplementary material for: Seed dressing with mefenpyr-diethyl as a safener for mesosulfuron-methyl application in wheat: The evaluation and mechanisms
Source: PLoS One. 2021 Aug 30;16(8):e0256884. doi: 10.1371/journal.pone.0256884 (PMC8405001; doi:10.1371/journal.pone.0256884)
Supplement: S2 Table — (DOCX) [file pone.0256884.s005.docx]

**S2 Table**. Primers used for qRT-PCR.

| Gene ID | Gene description | Primers(5’-3’) | |
| --- | --- | --- | --- |
| TraesCS2D01G197300 | GAPDH | Forward | GCGGCAAACATAGTAAAAGGA |
|  |  | Reverse | GATTCTGTTACAACTGGGGTGAT |
| TraesCS6B01G317400 | ALS1 | Forward | GTTGGATCAGCAGAAGAGGGA |
|  |  | Reverse | CGTGGCCGCTTGTAAGTGTA |
| TraesCS6D01G201500 | ALS2 | Forward | GCCTAACTCTGGAGGTAACTGG |
|  |  | Reverse | TTTTCACGCCTCAAAGCAAT |
| TraesCS5B01G259700 | GSTF1 | Forward | CGTGGAGAAGACGAAGAAGG |
|  |  | Reverse | TCCGCGAAGCTGAAGAAGT |
| TraesCS5B01G426400 | GSTU1B | Forward | CGCCGAGATGCTGGAGAT |
|  |  | Reverse | CCTCCGCCAGGCTGAAC |
| TraesCS4D01G238800 | AOX | Forward | AAGGCGGACATGAACATCG |
|  |  | Reverse | CGGGGCACTGCTTGTTGT |
| TraesCS7D01G404900 | CYP450 | Forward | TTCAAGGAGATCGTGCTGGA |
|  |  | Reverse | TTCATCATGTCGTCGAACCG |
| TraesCS3D01G120200 | UGT | Forward | AGCAACGACCTGTTCAAGCA |
|  |  | Reverse | TCCTTCCTCAGCACCCACA |
| TraesCS2D01G528500 | LPO | Forward | AGCGTGCTACACCCAATCC |
|  |  | Reverse | CATCTCCATGCCGTACTTGC |
| TraesCS2D01G107600 | POD | Forward | GCAACCTCAAGTCCCAGAAG |
|  |  | Reverse | GTTGCTTGCGAAGTTGTTGA |
| TraesCS5D01G464800 | WHAB1.6 | Forward | TTTGCCAAGAACCGTGAGC |
|  |  | Reverse | CCCATGAGCACGACCTGAC |
| TraesCS6B01G056800 | CAT | Forward | CCGTCATCGTCCGCTTCT |
|  |  | Reverse | ACTTGGGGTTGGGCTTGA |
| TraesCS5D01G480600 | ABCC4 | Forward | GCCGTATGGATGAGTTGCTT |
|  |  | Reverse | TCTCCTCCGTGTATGCTTAGTG |
| TraesCS7B01G149200 | HSP90 | Forward | CTGAGGGACACGAGCATGG |
|  |  | Reverse | GGTCTCGAACAGCAGCATCA |
