# Supplementary material for: Seed dressing with mefenpyr-diethyl as a safener for mesosulfuron-methyl application in wheat: The evaluation and mechanisms
Source: PLoS One. 2021 Aug 30;16(8):e0256884. doi: 10.1371/journal.pone.0256884 (PMC8405001; doi:10.1371/journal.pone.0256884)
Supplement: S3 Table — (DOCX) [file pone.0256884.s006.docx]

**S3 Table. Number of DEGs identified as transcription factors (TFs) and protein kinases (PKs).**

| TF**s** | | PK**s** | |
| --- | --- | --- | --- |
| Family | No. of DEGs | Family | No. of DEGs |
| AP2/ERF-AP2 | 2 | AGC_RSK-2 | 3 |
| AP2/ERF-ERF | 52 | Aur | 1 |
| AP2/ERF-RAV | 2 | CAMK_CAMKL-CHK1 | 9 |
| B3 | 2 | CAMK_CDPK | 7 |
| B3-ARF | 4 | CAMK_OST1L | 5 |
| bHLH | 35 | CK1_CK1 | 1 |
| bZIP | 14 | CMGC_CDK-CRK7-CDK9 | 1 |
| C2C2-CO-like | 8 | CMGC_CDKL-Os | 2 |
| C2C2-Dof | 8 | CMGC_MAPK | 1 |
| C2C2-GATA | 5 | Group-Pl-3 | 1 |
| C2C2-LSD | 3 | NEK | 4 |
| C2H2 | 22 | RLK-Pelle_CR4L | 2 |
| C3H | 10 | RLK-Pelle_CrRLK1L-1 | 5 |
| CPP | 1 | RLK-Pelle_DLSV | 51 |
| CSD | 2 | RLK-Pelle_Extensin | 6 |
| DBB | 3 | RLK-Pelle_L-LEC | 19 |
| DBP | 1 | RLK-Pelle_LRK10L-2 | 10 |
| FAR1 | 6 | RLK-Pelle_LRR-I-1 | 6 |
| GARP-G2-like | 12 | RLK-Pelle_LRR-III | 24 |
| GeBP | 1 | RLK-Pelle_LRR-V | 6 |
| GRAS | 11 | RLK-Pelle_LRR-VI-1 | 5 |
| HB-HD-ZIP | 16 | RLK-Pelle_LRR-VI-2 | 5 |
| HB-KNOX | 3 | RLK-Pelle_LRR-VII-1 | 1 |
| HB-other | 5 | RLK-Pelle_LRR-VIII-1 | 1 |
| HSF | 16 | RLK-Pelle_LRR-Xb-1 | 1 |
| LIM | 1 | RLK-Pelle_LRR-XI-1 | 19 |
| LOB | 8 | RLK-Pelle_LRR-XII-1 | 18 |
| MADS-MIKC | 2 | RLK-Pelle_LRR-XIV | 2 |
| MADS-M-type | 1 | RLK-Pelle_LysM | 1 |
| MYB | 47 | RLK-Pelle_PERK-1 | 7 |
| MYB-related | 19 | RLK-Pelle_RLCK-IV | 1 |
| NAC | 44 | RLK-Pelle_RLCK-IXa | 1 |
| NF-X1 | 3 | RLK-Pelle_RLCK-IXb | 3 |
| OFP | 4 | RLK-Pelle_RLCK-Os | 1 |
| PLATZ | 4 | RLK-Pelle_RLCK-V | 9 |
| TCP | 9 | RLK-Pelle_RLCK-VI | 5 |
| Trihelix | 7 | RLK-Pelle_RLCK-VIIa-2 | 7 |
| WRKY | 32 | RLK-Pelle_RLCK-VIIb | 3 |
| zf-HD | 4 | RLK-Pelle_RLCK-X | 3 |
|  |  | RLK-Pelle_RLCK-XII-1 | 1 |
|  |  | RLK-Pelle_RLCK-XIII | 4 |
|  |  | RLK-Pelle_RLCK-XV | 1 |
|  |  | RLK-Pelle_SD-2b | 21 |
|  |  | RLK-Pelle_WAK | 28 |
|  |  | RLK-Pelle_WAK_LRK10L-1 | 1 |
|  |  | STE_STE11 | 3 |
|  |  | TKL-Pl-4 | 4 |
| Total | 429 |  | 320 |
