# Supplementary material for: Seed dressing with mefenpyr-diethyl as a safener for mesosulfuron-methyl application in wheat: The evaluation and mechanisms
Source: PLoS One. 2021 Aug 30;16(8):e0256884. doi: 10.1371/journal.pone.0256884 (PMC8405001; doi:10.1371/journal.pone.0256884)
Supplement: S4 Table — (DOCX) [file pone.0256884.s007.docx]

**S4 Table. Enzymes related to DEGs in the most enriched pathways**

| **Treatment** | **Enriched Kegg pathway** | **Enzymes related to DEGs** |
| --- | --- | --- |
| **Safener** | Glutathione metabolism | glutathione peroxidase [EC:1.11.1.9] |
|  |  | glutathione S-transferase [EC:2.5.1.18] |
|  | alpha-Linolenic acid metabolism | lipoxygenase [EC:1.13.11.12] |
|  |  | hydroperoxide dehydratase [EC:4.2.1.92] |
|  |  | 12-oxophytodienoic acid reductase [EC:1.3.1.42] |
|  | Linoleic acid metabolism | lipoxygenase [EC:1.13.11.12] |
|  |  | linoleate 9S-lipoxygenase [EC:1.13.11.58] |
|  | Phenylpropanoid biosynthesis | cinnamyl-alcohol dehydrogenase [EC:1.1.1.195] |
|  |  | peroxidase [EC:1.11.1.7] |
|  |  | beta-glucosidase [EC:3.2.1.21] |
|  |  | cinnamoyl-CoA reductase [EC:1.2.1.44] |
| **Herbicide** | Starch and sucrose metabolism | UDPglucose 6-dehydrogenase [EC:1.1.1.22] |
|  |  | sucrose synthase [EC:2.4.1.13] |
|  |  | 1,4-beta-D-xylan synthase [EC:2.4.2.24] |
|  |  | hexokinase [EC:2.7.1.1] |
|  |  | glucose-1-phosphate adenylyltransferase [EC:2.7.7.27] |
|  |  | pectinesterase [EC:3.1.1.11] |
|  |  | trehalose 6-phosphate phosphatase [EC:3.1.3.12] |
|  |  | beta-amylase [EC:3.2.1.2] |
|  |  | endoglucanase [EC:3.2.1.4] |
|  |  | polygalacturonase [EC:3.2.1.15] |
|  |  | beta-fructofuranosidase [EC:3.2.1.26] |
|  |  | UDP-glucuronate 4-epimerase [EC:5.1.3.6] |
|  |  | alpha-1,4-galacturonosyltransferase [EC:2.4.1.43] |
|  |  | beta-D-xylosidase 4 [EC:3.2.1.37] |
|  |  | trehalose 6-phosphate synthase/phosphatase [EC:2.4.1.15] 3.1.3.12] |
|  | Phenylalanine metabolism | primary-amine oxidase [EC:1.4.3.21] |
|  |  | peroxidase [EC:1.11.1.7] |
|  |  | tyrosine aminotransferase [EC:2.6.1.5] |
|  |  | 4-coumarate--CoA ligase [EC:6.2.1.12] |
|  |  | phenylalanine ammonia-lyase [EC:4.3.1.24] |
|  |  | phenylalanine/tyrosine ammonia-lyase [EC:4.3.1.25] |
|  | Phenylpropanoid biosynthesis | cinnamyl-alcohol dehydrogenase [EC:1.1.1.195] |
|  |  | beta-glucosidase [EC:3.2.1.21] |
|  |  | Ferulate-5-hydroxylase [EC:1.14.-.-] |
|  |  | serine carboxypeptidase-like 19 [EC:3.4.16.- 2.3.1.91] |
|  |  | coniferyl-aldehyde dehydrogenase [EC:1.2.1.68] |
| **Herbicide**  **+Safener** | Starch and sucrose metabolism | starch phosphorylase [EC:2.4.1.1] |
|  |  | 1,4-beta-D-xylan synthase [EC:2.4.2.24] |
|  |  | pectinesterase [EC:3.1.1.11] |
|  |  | beta-amylase [EC:3.2.1.2] |
|  |  | beta-glucosidase [EC:3.2.1.21] |
|  |  | beta-fructofuranosidase [EC:3.2.1.26] |
|  |  | beta-glucosidase [EC:3.2.1.21] |
|  |  | UDP-glucuronate 4-epimerase [EC:5.1.3.6] |
|  |  | trehalose 6-phosphate synthase/phosphatase [EC:2.4.1.15]  3.1.3.12] |
|  | Sulfur metabolism | adenylyl-sulfate reductase (glutathione) [EC:1.8.4.9] |
|  |  | serine O-acetyltransferase [EC:2.3.1.30] |
