# Supplementary material for: Seed dressing with mefenpyr-diethyl as a safener for mesosulfuron-methyl application in wheat: The evaluation and mechanisms
Source: PLoS One. 2021 Aug 30;16(8):e0256884. doi: 10.1371/journal.pone.0256884 (PMC8405001; doi:10.1371/journal.pone.0256884)
Supplement: Attachment — Submitted filename: Response to Reviwers.doc [file pone.0256884.s009.doc]

Dear Editor and Reviewer:

Thank you for your letter and for the reviewer’s comments concerning our manuscript entitled “**Seed dressing with mefenpyr-diethyl as a safener for mesosulfuron-methyl application in wheat: the evaluation and mechanisms.**” (PONE-D-21-06771R1). Those comments are all valuable and very helpful for revising and improving our paper, as well as of important guiding significance to our researches. We have studied the comments carefully and made corrections which we hope meet with approval. Revised portions are marked under track changes mode in MS. The main corrections in the paper and the responds to the reviewer’s comments are as following:

Please NOTE: the red words are comments or questions from the editor or reviewer, and the black words are our replies.

**Reviewer #2:**

1. Line 38: need to rephrase “yield loss of production in wheat producing regions”.

Thanks for your suggestion. We have rephrased this sentence by deleting the unnecessary words.

2. Line 147: pay attention to the format of “2-∆∆Ct”

Thank you for your reminding. The “-∆∆Ct” has been set in upper superscrip format.

3. Line 172: delete “of plants”.

Thank you for your suggestion. The phrase “of plants” has been deleted to make the sentence concise.

4. Line 226: lowercase for “L” in “alpha-Linolenic acid metabolism”.

Thank you for your reminding. The word “Linolenic” has been changed to lowercase.

5. Line 250: check the spelling “mephenpr-diethyl”.

Thank you for your reminding. The word “mephenpr-diethyl” has been changed to “mefenpyr-diethyl” throughout the manuscript.

6. Line 257: check the spelling “WORKY”

Thank you for your reminding. “WORKY” has been changed to “WRKY”.

7. Line 278: check the style of citation “Duhoux et al. (2017)” throughout the manuscript, which differ to line 59,100,118,300,318……

Thank you for your reminding. The style of citation “Duhoux et al. (2017)” has been unified.

8. Line 283: suggest change “researches” to “studies”

Thank you for your suggestion. It has been changed now.

9. Line 320: need to rephrase the sentence “……could be evidence.”

Thank you for your reminding. We have rephrased this sentence.

10. Line 355: unified the citation of “S2 Fig” or “Fig S3”, and table?

Thank you for your reminding. We have checked all the format of the supplementary Figs and tables and unified them.

11. Line 378: change “enzymic” to “enzymatic”.

Thank you for your suggestion. It has been corrected.

We tried our best to improve the manuscript and made some changes in the manuscript, including those in the format of the references list. These changes will not influence the content and framework of the paper. And here we did not list the changes but marked in the revised paper.

We appreciate for Editors/Reviewers’ warm work earnestly, and hope that the correction will meet with approval.

Once again, thank you very much for your comments and suggestions. If you have any question, please contact us. I am looking forward to hearing from you.

Best regards and wishes!

Respectfully submitted,

Libing Yuan (Ph.D.)

College of Plant Protection, Hebei Agricultural University

Plant Protection Institute, Hebei Academy of Agricultural and Forestry Sciences, P.R. China

Tel. /Fax: +86-312-5915162.

E-mail:yuanlibing83@163.com
